# Genetic variation in bovine LAP3 and SIRT1 genes associated with fertility traits in dairy cattle

**DOI:** 10.1186/s12863-024-01209-x

**Published:** 2024-03-18

**Authors:** Destaw Worku, Archana Verma

**Affiliations:** 1 Department of Animal Science, College of Agriculture, Food and Climate Science, Injibara University, Injibara, Ethiopia; 2 Animal Genetics and Breeding Division, ICAR -National Dairy Research Institute, Karnal, India

**Keywords:** LAP3, SIRT1, SNPs, Fertility traits, Association analysis, Dairy cattle

## Abstract

**Background:**

The genetic progress of fertility and reproduction traits in dairy cattle has been constrained by the low heritability of these traits. Identifying candidate genes and variants associated with fertility and reproduction could enhance the accuracy of genetic selection and expedite breeding process of dairy cattle with low-heritability traits. While the bovine LAP3 and SIRT1 genes exhibit well-documented associations with milk production traits in dairy cattle, their effect on cow fertility have not yet been explored. Eleven single nucleotide polymorphisms (SNPs), comprising five in the promoter (rs717156555: C > G, rs720373055: T > C, rs516876447: A > G, rs461857269: C > T and rs720349928: G > A), two in 5’UTR (rs722359733: C > T and rs462932574: T > G), two in intron 12 (rs110932626: A > G and rs43702363: C > T), and one in 3’UTR of exon 13 (rs41255599: C > T) in LAP3 and one in SIRT1 (rs718329990:T > C) genes, have previously been reported to be associated with various traits of milk production and clinical mastitis in Sahiwal and Karan Fries dairy cattle. In this study, the analysis primarily aimed to assess the impact of SNPs within LAP3 and SIRT1 genes on fertility traits in Sahiwal and Karan Fries cattle. Association studies were conducted using mixed linear models, involving 125 Sahiwal and 138 Karan Fries animals in each breed. The analysis utilized a designated PCR-RFLP panel.

**Results:**

In the promoter region of the LAP3 gene, all variants demonstrated significant (*P* < 0.05) associations with AFC, except for rs722359733: C > T. However, specific variants with the LAP3 gene’s promoter region, namely rs722359733: C > T, rs110932626: A > G, rs43702363: C > T, and rs41255599: C > T, showed significant associations with CI and DO in Sahiwal and Karan Fries cows, respectively. The SNP rs718329990: T > C in the promoter region of SIRT1 gene exhibited a significant association with CI and DO in Sahiwal cattle. Haplotype-based association analysis revealed significant associations between haplotype combinations and AFC, CI and DO in the studied dairy cattle population. Animals with H2H3 and H2H4 haplotype combination exhibited higher AFC, CI and DO than other combinations.

**Conclusions:**

These results affirm the involvement of the LAP3 and SIRT1 genes in female fertility traits, indicating that polymorphisms within these genes are linked to the studied traits. Overall, the significant SNPs and haplotypes identified in this study could have the potential to enhance herd profitability and ensure long-term sustainability on dairy farms by enabling the selection of animals with early age first calving and enhance reproductive performance in the dairy cattle breeding program.

**Supplementary Information:**

The online version contains supplementary material available at 10.1186/s12863-024-01209-x.

## Background

The success of dairy production depends on the enhanced productive and reproductive performance of animals. Within the dairy industry, optimal fertility plays a crucial role in driving profitability for farmers. In previous decades, the breeding programs predominantly aimed at enhancing milk production, but unfortunately, attention to cow fertility has been neglected, resulting in a decrease in fertility. Therefore, it is essential to incorporate fertility traits into breeding to either enhance fertility or halt the genetic downward trend [1]. Various phenotypic measures including age at first calving, timing of estrus, pregnancy rate, days open, days to first service, services per conception and calving interval, play pivotal in female fertility within breeding programs [2, 3]. In this context, systematically selecting for these phenotypes is imperative to prevent the decline in fertility observed in dairy cattle. However, the unfavourable genetic correlation between milk production and fertility, as noted in Pryce et al. [4], presents a challenge. Th higher demands of increased milk production lead to a greater negative energy balance, resulting in lower responsiveness to genetic progress through selective breeding due to their low heritability, as discussed in Ghiasi et al. [5]. This combination makes the selection process more challenging.

In the last decade, the introduction of genomic selection into the dairy cattle improvement program has provided a more comprehensive measure of progress and contribute to stabilize and recover the genetic trends of reproductive traits in dairy cattle, particularly those with low heritability [6, 7]. Therefore, gaining a deeper insight into the biological mechanisms and genetic architecture of fertility traits has the potential to improve the accuracy of genetic selection and expedite the breeding process of dairy cattle. This prompts the need to explore candidate genes or known causal variants associated with economic traits, including fertility traits, in dairy cattle.

The candidate gene approach, focusing on selecting informative markers associated with fertility traits, can be applied in breeding programs through marker-assisted selection (MAS), particularly when the heritability of the trait is low. In this context, researchers are actively working to pinpoint potential candidate genes and / or causal variants known to be associated with fertility traits in cattle [8–11]. In the last few years, several studies have explored the genetic variations of bovine leucine amino peptidase three (LAP3) and silent information regulator one (SIRT1) genes associated with milk production and health related traits in dairy cattle [12–16].

Leucine amino peptidase belong to either the M1 or M17 family of peptidases and are commonly recognized as cell maintenance enzymes that play a critical role in the turnover of peptides. In animals, the LAP3 gene, located on bovine chromosome 6, is implicated in MHCI antigen presentation, the osmotic regulation of blood pressure, and various physiological functions, as well as cataract formation [17, 18]. Additionally, LAP3 has been proposed to play a part in vesicle trafficking, influencing glucose-transport, as well as the catabolism of oxytocin and vasopressin. Consequently, LAPs and LAP related aminopeptidases may exert a variety of functions and have significant impacts on cellular and physiological activities. Moreover, substantial studies on cattle have reported numerous promising mutations in the LAP3 gene that explain genetic variations in traits such as milk production, somatic cell score, and clinical mastitis in dairy cattle [12–16], as well as birth weight in sheep [19]. LAP3 has also been identified as a candidate gene affecting the weight of the longissimus dorsi muscle in Huaxi cattle [20], milk traits [21] and direct calving ease [22] in cattle. For dairy cattle, a QTL affecting milk production traits was pinpointed on bovine chromosome 6, positioned between the potential candidate genes ABCG2 and LAP3 [23–25]. The involvement of LAP3 as a candidate gene that affects important production traits such as visceral organ weight, body size, and carcass traits was supported by the previous authors [26–29].

The bovine SIRT1 gene, located on chromosome 28, is involved in regulating physiological functions across various tissues, including liver, muscle, pancreas, testis, ovaries and adipose tissue. In mammals, SIRT1 has evolved to modify the activity of a growing number of transcription factors, such as p53, NF-κB, and PGC-1α, suggests that SIRT1 plays a crucial role in diverse cellular responses to stress, inflammation, and nutrients [30]. Previous studies on polymorphisms have identified several significant variants in SIRT1 gene associated with body size traits [31], intramuscular fat content [32] and carcass traits [33] in cattle. Recently, it was noted that, the g.-274 C > G variant in the SIRT1 gene has a simultaneous impact on both milk production and reproductive performance traits in the Agerolese cattle breed [34].

Based on the above evidence, we hypothesized that variations within the LAP3 and SIRT1 genes may play a role in the fertility traits of dairy cattle. Although the effects of LAP3 and SIRT1 variants on milk production and growth traits have been previously documented, their effects on fertility traits in Sahiwal and Karan Fries cows have not yet been studied. Therefore, this study aimed to verify the genetic effects of LAP3 and SIRT1 gene variants on age at first calving (AFC), calving interval (CI) and days open (DO) in Sahiwal and Karan Fries dairy cattle.

## Materials and methods

### Animals and phenotypic data collection

The present study was conducted on a randomly selected 125 Sahiwal cows from 28 sire families and 138 Karan Fries (Holstein Friesian x Tharparkar) cows from 47 sire families, with all parities maintained at the ICAR-National Dairy Research Institute (ICAR-NDRI), Karnal, India. Lactating Sahiwal and Karan Fries cows used for the study were bred by artificial insemination techniques with frozen thawed semen from registered bulls. Comprehensive herd information, encompassing details such as animal, sire, and dam ID, birth date, insemination date, calving date, dry-off date, days in milk and lactation performance were extracted from the history-cum pedigree records. The phenotypic data pertinent to records were then obtained, and from these, the traits AFC, CI and DO were derived and subsequently used in the analysis. Descriptive statistics of the phenotypic values for fertility traits of data set used for fertility traits analysis is presented in Table S1.

### Blood samples, DNA extraction and SNP genotyping

Blood samples were aseptically collected from the jugular vein of 125 Sahiwal and 138 Karan Fries cattle with ethical considerations. The collection utilized 0.5 ml EDTA as an anticoagulant, and the samples were then stored at -20ºC. Post-collection, animals were safely released back into the herd. Genomic DNAs were subsequently extracted from the whole blood samples using phenol-chloroform extraction, following standard protocols [35]. The quality and quantity of DNA was assessed by agarose gel-electrophoresis and spectrophotometer. The sets of PCR primers used to amplify the targeted regions of LAP3 and SIRT1 genes, PCR amplification protocols and SNP genotyping methods were similar to those described in our previous work [12–14]. The genotype data obtained from 125 Sahiwal and 138 Karan Fries cows were used for single marker analysis. Furthermore, the genotype data of 220 cows (110 Sahiwal and 110 Karan Fries cows) were deemed suitable for performing association analysis between haplotype combinations of SNPs in the promoter region of the LAP3 gene and fertility traits.

### Statistical analysis

The extent of linkage disequilibrium (LD) between the identified SNPs and haplotype construction analysis were performed by using the Haploview 4.2 software [36] and SNPstat online server [37], respectively. The association analysis between SNP genotypes and /or haplotype combinations with each fertility trait was evaluated using mixed model procedure of SAS 9.2 [38] with genotyped individual animal included as random effect.

For AFC, breed, year of birth, season of birth and genotypes were included as fixed effects in the model. For CI and DO, breed, year of calving, season of calving, parity and genotypes included as fixed effects in the model. Year of birth and year of calving were divided into 5-yearly intervals. Likewise, due to the limited number of observations and the strong correlation observed between parity 5 and the subsequent lactations, all parities beyond 5 were pooled under parity 5.

The following full statistical models were used for analysis:

Y_*ijklm*_ = µ + α_*i*_ + g_*j*_ + b_*k*_ +y_*l*_ + s_*m*_ + e_*ijklm*_ (1)

Y_*ijklmn*_ = µ + α_*i*_ + g_*j*_ + b_*k*_ +y_*l*_ + s_*m*_ +l_n_ + e_*ijklmn*_ (2)

where Y_*ijklm*_ is the phenotypic value of the trait of interest for the *i*^th^ cow; µ is the overall mean; α_*i*_ is the random animal effect, g_*j*_ is the fixed effect of SNP genotype (*j* = 0, 1 or 2 for homozygote allele 1, heterozygote, and homozygote allele 2) or haplotype combination effect (*j* = 1,…0.8), b_*k*_ is the fixed effect of breed (k = 1 and 2), y_*l*_ is the fixed effect of calving and / or birth year (1–3), s_*m*_ is the fixed effect of calving and / or birth season (1: December - March; 2: April - June; 3: July - September and 4: October - November), l_n_ is the fixed effect of parity (k = 1,…5; for CI and DO only) and e_*ijklm*_ is the random residual effect. Least square means for each genotype were anayzed using the lsmeans package in R [39], and Tukey’s pairwise mean comparison was set to determine differences between allele combinations. Results were expressed as least squares means ± standard error. Effects associated with *P* values < 0.05 were considered significant. Furthermore, the additive (a), dominant (d), and substitution (α) effects were calculated using the following formulas: a = $\frac{AA-BB}{2}$; d = $AB-\frac{\left(AA-BB\right)}{2}$; α = a + d (q-p) [40], where AA, BB, and AB are the least square means of fertility traits in the respective genotypes, and p and q were the frequencies of allele A and B, respectively.

## Results

### Associations between SNP genotypes and fertility traits

All identified SNPs, along with their genotypic and allelic frequencies were described in our previous study [12–14]. In this analysis, we investigated the associations between ten SNPs in LAP3 and one SNP in SIRT1 and fertility traits in dairy cattle (Table 1). Other non-genetic factors such as breed, season and year of birth were incorporated into the model for AFC, while breed, season and year of calving and parity were included in the model for CI and DO.

The association analysis revealed significant associations (*p* < 0.05) between five promoter SNPs (rs71715655: C > G, rs720373055: T > C, rs516876447: A > G, rs461857269: C > T, rs720349928: G > A) and one 5’UTR SNP (rs462932574: T > G) in the LAP3 gene and AFC in the studied population (Table 2). Referring to genotypes of these SNPs, heterozygous animals exhibited a significantly longer AFC compared to other genotypes. However, there were no significant associations (*p* > 0.05) between these LAP3 variants and CI and DO among animals. On the contrary, the SNP rs722359733: C > T located in the 5’UTR of LAP3 strongly associated with CI (*p* value = 0.00145) and DO (*p* value = 0.00640) (Table 2), whereas AFC was not affected by this SNP genotypes (*p* > 0.05) in the studied population. Results indicate that animals with genotype TT showed significantly shorter CI and DO than cows with CT genotype. Significant differences were also observed between alternative homozygotes: animals with genotype TT had shorter CI and DO than cows with CC genotype.

In the intron 12 region of LAP3, the SNPs rs110932626: A > G and rs43702363: C > T were significantly associated with CI and DO in Karan Fries cows (*p* values ≤ 0.015; Table 3). Heterozygote genotypes of these SNPs (rs110932626: A > G and rs43702363: C > T) in Karan Fries cows were associated with shorter CI and DO compared to GG and TT genotypes (Table 3). In the context of exon 13 of LAP3, a 3’UTR variant (rs41255599: C > T) was significantly associated with CI (*p* value = 0.0293) and DO (*p* value = 0.0243) in Sahiwal cows (Table 3). Further, the additive, dominant and substitution effects of the ten SNPs are presented in Table S2.

Regardless of associations with polymorphisms in the SIRT1 gene, a notable promoter variant (rs718329990: T > C) significantly associated with CI (*p* value = 0.0005) and DO (*p* value = 0.0021) in Sahiwal cows (Table 4). It was found that, genotype TT had shorter CI and DO than their counterparts. Further, the additive, dominant and substitution effects of one SNP is presented in Table S2.

**Table 1:** List of the 11 SNPs obtained from the previous polymorphism association studies in Sahiwal and Karan Fries cattle

Gene	SNP ID	Region	Location (Chr: bp)	Mutation
LAP3	rs717156555	Promoter	6:37140485	C > G
	rs720373055	Promoter	6:37140513	T > C
	rs516876447	Promoter	6:37140523	A > G
	rs461857269	Promoter	6:37140644	C > T
	rs720349928	Promoter	6:37140681	G > A
	rs722359733	5’UTR	6:37140767	C > T
	rs462932574	5’UTR	6:37140802	T > G
	rs110932626	Intron 12	6:37165306	A > G
	rs43702363	Intron 12	6:37165545	C > T
	rs41255599	3’UTR	6:37166157	C > T
SIRT1	rs718329990	Promoter	28:24405202	T > C

**Table 2:** Associations between SNPs genotypes in the promoter and 5’UTR region of LAP3 gene and fertility traits in dairy cattle

		Least-square means ± standard error		
SNP	Genotype (No.)	AFC (days)	CI (days)	DO (days)
rs717156555: C > G	CC (266)	1129.00 ± 22.00^b^	464.12 ± 12.29	183.38 ± 12.24
	CG (104)	1195.00 ± 29.10^a^	475.56 ± 15.54	188.99 ± 15.52
	*p*	**0.0128** ^*^	0.4516	0.7108
rs720373055: T > C	TT (266)	1129.00 ± 22.00^b^	458.00 ± 12.10	173.05 ± 11.50
	TC (104)	1195.00 ± 29.10^a^	472.00 ± 15.00	182.55 ± 14.20
	*p*	**0.0128** ^*^	0.4218	0.6636
rs516876447: A > G	AA (105)	1087.00 ± 32.10^b^	462.00 ± 16.40	177.00 ± 16.10
	AG (132)	1148.00 ± 26.70^a^	463.00 ± 14.10	173.00 ± 13.30
	GG (133)	1202.00 ± 31.80^a^	461.00 ± 16.40	179.00 ± 15.50
	*p*	**0.0341** ^*^	0.9668	0.9613
rs461857269: C > T	CC (266)	1129.00 ± 22.00^b^	458.00 ± 12.10	173.00 ± 11.50
	CT (104)	1195.00 ± 29.10^a^	472.00 ± 15.00	182.00 ± 14.20
	*p*	**0.0288** ^*^	0.4218	0.6636
rs720349928: G > A	GG (266)	1129.00 ± 22.00^b^	458.00 ± 12.10	173.00 ± 11.50
	GA (104)	1195.00 ± 29.10^a^	472.00 ± 15.00	182.00 ± 14.20
	*p*	**0.0288** ^*^	0.4218	0.6636
rs722359733: C > T	CC (225)	1129.00 ± 25.10	458.00 ± 12.30^ab^	174.00 ± 11.60^b^
	CT (101)	1176.00 ± 34.40	504.00 ± 18.40^a^	208.00 ± 17.60^a^
	TT (44)	1210.00 ± 53.30	429.00 ± 22.20^b^	143.00 ± 21.20^c^
	*p*	0.2783	**0.00145** ^**^	**0.00640** ^**^
rs462932574: T > G	TT (266)	1129.00 ± 22.00^b^	463.87 ± 12.23	173.05 ± 11.50
	TG (104)	1195.00 ± 29.10^a^	475.95 ± 15.43	182.00 ± 14.20
	*p*	**0.0288** ^*^	0.4218	0.6636

**Table 3:** Associations between SNPs genotypes in intron 12 and exon 13 region of LAP3 gene and fertility traits in Karan Fries and Sahiwal cattle

			Least-square means ± standard error		
SNP	Breed	Genotype (No.)	AFC (days)	CI (days)	DO (days)
rs110932626: A > G	Karan Fries	AG (146)	1085.00 ± 22.20	469.00 ± 9.94^b^	183.00 ± 9.06^b^
		GG (157)	1089.00 ± 21.50	498.00 ± 10.50^a^	217.00 ± 9.57^a^
		*p*	0.913	**0.0150** ^*^	**0.00161** ^**^
rs43702363: C > T	Karan Fries	CT (151)	1082.00 ± 22.80	470.00 ± 9.60^b^	183.00 ± 8.73^b^
		TT (152)	1092.00 ± 21.20	502.00 ± 11.00^a^	222.00 ± 9.99^a^
		*p*	0.741	**0.00662** ^**^	**0.000356** ^***^
rs41255599:C > T	Sahiwal	CC (56)	1296.44 ± 39.73	434.29 ± 19.51^b^	149.00 ± 18.35^b^
		CT (91)	1194.79 ± 33.80	479.00 ± 16.01^a^	191.00 ± 15.01^a^
		TT (102)	1247.13 ± 33.21	434.00 ± 16.41^b^	146.00 ± 15.48^b^
		*p*	0.1168	**0.0293** ^*^	**0.0243** ^*^

**Table 4:** Association between SNP genotypes in promoter region of SIRT1 gene and fertility traits in Sahiwal dairy cattle

		Least-square means ± standard error		
SNP	Genotype (No.)	AFC (days)	CI (days)	DO (days)
rs718329990: T > C	TT (63)	1220.00 ± 36.70	402.24 ± 18.30^b^	123.35 ± 17.80^b^
	TC (112)	1213.00 ± 29.70	478.03 ± 13.70^a^	190.39 ± 13.40^a^
	CC (26)	1324.00 ± 50.10	463.00 ± 27.20^ab^	163.00 ± 26.80^ab^
	*p*	0.0963	**0.0005** ^***^	**0.0021** ^**^

### Association between haplotype combinations and fertility traits

**Fig. 1:**
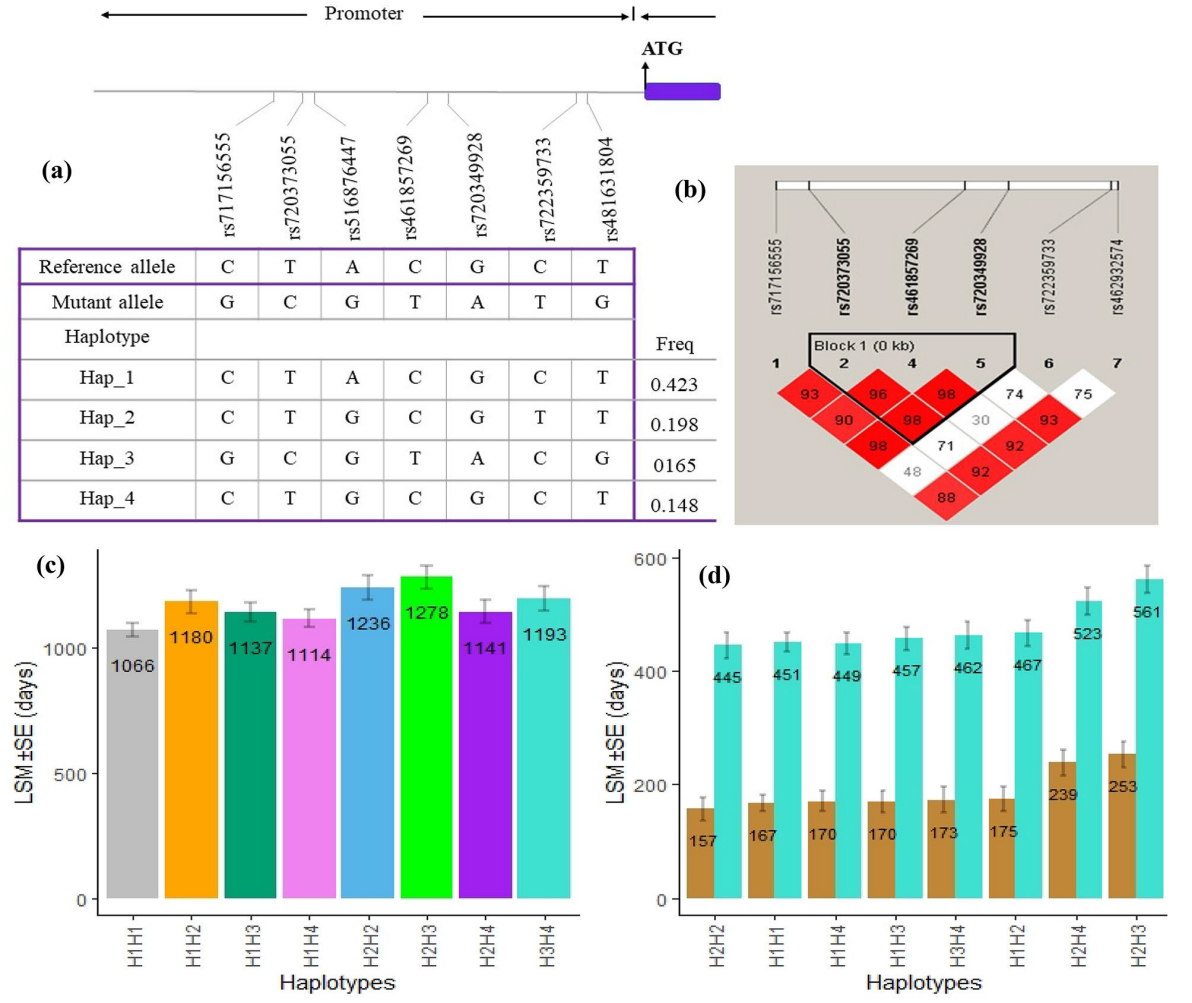
Genetic variations and haplotype analysis of the LAP3 gene in dairy cattle. (**a**) Schematic representation of the list and location of single nucleotide polymorphisms (SNPs) sites and major haplotype analysis inferred across seven SNPs in the promoter region of LAP3 gene. ATG: Transcription start site. Box filled with purple is coding and non-coding regions. Numbers at the bottom indicate SNP ID, while table shows the nucleotide sequence of SNPs present in Hap_1 (haplotype 1), Hap_2 (haplotype 2), Hap_3 (haplotype 3) and Hap_4 (haplotype 4) of LAP3 gene with their population frequency (Freq: frequency) at the right. (**b)** Pairwise linkage disequilibrium (LD) analysis of SNPs located on promoter region of LAP3 gene. There exists one LD block composed of three SNPs and block definitions are based on the Gabriel et al. default criteria. The values in the boxes are pairwise SNP correlations (**D**). (**c)** Bar plots for least square mean values contrasting different haplotype combinations with AFC trait in dairy cattle. Error bars represent standard error. Numbers inside the columns indicate least square mean values of each haplotype combination for the trait. (**d)** Bar plots for least square mean values contrasting different haplotype combinations with CI and DO traits in dairy cattle. Error bars represent standard error. Numbers inside the columns indicate least square mean values of each haplotype combination for the traits

For association analysis of haplotype combinations of LAP3 genotypes in the promoter region and fertility traits, genetic polymorphisms prevalent in both the studied populations (Sahiwal and Karan Fries) with frequencies greater than 5% were included (Fig. 1a). The degree of linkage disequilibrium (LD) among the seven identified promoter variants in the LAP3 gene was estimated using Haploview 4.2, revealed the presence of one haplotype block encompassing three of the SNPs (Fig. 1b). Seven of the SNPs in the promoter region of the LAP3 gene, prevalent in both Sahiwal and Karan Fries dairy cattle, were used for haplotype reconstruction. Haplotypes with frequencies greater than 5% were retained for association analysis, revealed four haplotypes: H1(CTACGCT), H2 (CTGCGTT), H3 (GCGTACG) and H4 (CTGCGCT), with frequencies of 0.423, 0.198, 0.165 and 0.148, respectively (Fig. 1a). These SNPs produced eight combined haplotypes: H1H1 (CC-TT-AA-CC-GG-CC-TT), H1H2 (CC-TT-AG-CC-GG-CT-TT), H1H3 (CG-TC-AG-CT-GA-CC-TG), H1H4.

(CC-TT-AG-CC-GG-CC-TT), H2H2 (CC-TT-GG-CC-GG-TT-TT), H2H3 (TC-GG-CT-GA-CT-TG), H2H4 (TT-GG-CC-GG-CT-TT) and H3H4 (CG-TC-GG-CT-GA-CC-TG). Statistical analysis revealed significant correlation between haplotype combinations and fertility traits, AFC (*p* value = 0.0006), CI (*p* value = 0.00021) and DO (*p* value = 0.0020) (Table S3). Cows carrying haplotype combination H2H3 (TC-GG-CT-GA-CT-TG) exhibited the prolonged days of AFC (Fig. 1c), CI and DO (Fig. 1d) in comparison with the other haplotype combinations.

## Discussion

In the past decades, intensive selection for increased milk production in dairy cattle has inadvertently led to a negative impact on cow fertility. Identifying dairy cows with optimal fertility would significantly enhance the overall profitability of dairy farming. However, fertility in dairy cattle is a complex trait, thus, identifying the likely causative genes and /or variants for fertility traits and incorporating them into marker-assisted selection (MAS) is challenging. If alleles with a moderate to large effect on fertility can be identified, implementing such alleles into breeding programs through MAS holds promise. Moreover, exploring genetic variants associated with fertility traits through a candidate gene approach contributes to unravelling the genetic basis of fertility in dairy cattle. Our previous studies in dairy cattle unveiled SNP variants in LAP3 and SIRT1 genes that affected the estimated breeding value of milk production traits and clinical mastitis in Sahiwal and Karan Fries dairy cattle [12–14]. This study sought to determine whether the polymorphisms in the LAP3 and SIRT1 genes, previously identified for milk production traits [12–14], were associated with fertility traits in dairy cattle. In this study, nearly the same SNPs as in our previous work were employed to perform an association analysis.

The results of this study indicated a significant genetic association between the SNPs and haplotype combinations of these genes and fertility traits. Notably, significant associations were found between almost all individual SNPs in the promoter and 5’UTR of LAP3 with AFC in dairy cows in the studied population. Specifically, the SNPs rs720373055: T > C and rs720349928: G > A in the promoter region of the LAP3 gene, associated with AFC in the current study, were found to generate and alter transcription factor binding sites, potentially playing a role in regulating transcription factors associated with improved lactation performance in dairy cattle [35]. These transcription factors could provide valuable molecular insights into the age-dependent regulatory effect of the LAP3 gene in cattle. However, the SNP rs722359733: C > T in LAP3, significantly associated with CI and DO in this study, has not been previously associated with milk yield traits for the same breed [12]. Therefore, it might be plausible to select for CI and DO without compromising the milk yield of dairy cows.

The SNPs rs110932626: A > G and rs43702363: C > T in the intron 12 region of LAP3 gene were found to be associated with CI and DO in Karan Fries cows. Particularly, Karan Fries cows expressing GG and TT genotypes tended to have prolonged CI and DO compared to those with AG and CT genotypes, respectively. In line with these findings, a study by Worku et al. [13] reported a similar tendency for the GG and TT genotypes associated with increased 305-day milk yield in Karan Fries cattle. Regarding polymorphisms in exon 13 (3’UTR) of LAP3, the variant rs41255599: C > T exhibited significant associations with both CI and DO in Sahiwal cows. Consistent with the associations explored in this study, Worku et al. [13] reported a significant association between rs41255599: C > T and milk production traits and clinical mastitis in Sahiwal cattle. Notably, the CT genotype showed a tendency to associate with increased CI and DO in Sahiwal cows. Similarly, Sahiwal cows heterozygous for the SNP rs41255599: C > T showed increased lactation performance compared to their counterparts [13]. These results serve as a compelling example illustrating the effect of variants in un favourable direction. Thus, selection for such variants for enhanced fertility could potentially compromise milk production traits in dairy cattle.

The existence of variants with substantial effects on complex quantitative traits appears to be more common in livestock [41]. This existence may be attributed, in part, to these variants being subjected to robust directional selection, and hence these variants should be subjected to balancing selection. In addition, a significant relationship was identified between haplotype combinations of LAP3 variants and fertility traits, with cows carrying haplotype combination H2H3 exhibited longer days of AFC, CI and DO compared to other haplotype combinations. It is noteworthy that the analysis of haplotypes composed of SNPs could provide more accurate information than analysing individual SNPs for economic trait association, capturing ancestral structure in the distribution of haplotypes [42]. As depicted in Fig. 1a and b, haplotypes consist of two or more polymorphic SNPs of the haploid sequences jointly inherited as a unit, providing a more comprehensive perspective. Using haplotypes has been reported to considerably increase the prediction accuracy of quantitative traits with low heritability compared to individual SNP markers [43–45].

In this study, the variant rs718329990: T > C within the SIRT1 gene was found to have a noteworthy and significant effect on the fertility of Sahiwal cows. Cows with the TT genotype exhibited shorter CI and DO than those with TC and CC genotypes, respectively. On the same note, cows with the TT genotype associated with decreased lactation performance in Sahiwal dairy cattle [13]. This may be attributed to the pleiotropic effects of the gene on multiple milk production and fertility traits in dairy cattle. There was also a reported probable signature of positive selection near the SIRT1 gene in the Yaroslavl cattle breed [21].

In light of all these findings, the significant genetic variations of the LAP3 and SIRT1 genes in this study are likely candidates for influencing production and fertility traits. Overall, these data collectively suggest that LAP3 and SIRT1 genes are involved in a variety of functions and their polymorphisms are significantly associated with milk production and fertility traits in dairy cattle. However, the specific molecular mechanism needs further investigation.

## Conclusion

This study has confirmed the genetic effects of LAP3 and SIRT1 genes on the fertility traits of dairy cattle. Notably, the SNP rs722359733: C > T, which is significantly associated with cow fertility and does not negatively affect milk production traits, could be incorporated into commercial SNP arrays. This inclusion has the potential to improve the accuracy of genomic estimated breeding values for fertility without compromising milk production traits in dairy cattle. Furthermore, animals with the H2H3 haplotype combination in the LAP3 gene associated with extended days of AFC, CI and DO, and selection of animals against this haplotype combination will likely to improve the reproductive performance of dairy cattle. This study contributes new evidence, urging further functional verification of polymorphisms in LAP3 and SIRT1 genes and offered new target genes for MAS in breeding dairy cattle for optimal fertility, thereby supporting sustainable dairy development. However, further association studies involving larger population sizes and functional verification are warranted to confirm the potential importance of these variants in the selection for improved reproductive performance in dairy cattle.

### Electronic supplementary material

Below is the link to the electronic supplementary material.

Supplementary Material 1

## Data Availability

The datasets generated and/or analysed during the current study are available in the NCBI-GenBank® repository with accession numbers (ON764231-ON764241).
