# Supplementary material for: Genetic variation in bovine LAP3 and SIRT1 genes associated with fertility traits in dairy cattle
Source: BMC Genom Data. 2024 Mar 18;25:32. doi: 10.1186/s12863-024-01209-x (PMC10949778; doi:10.1186/s12863-024-01209-x)
Supplement: Supplementary file 1 — Supplementary Material 1 [file 12863_2024_1209_MOESM1_ESM.docx]

**Table S1** Descriptive statistics for the phenotypic values of fertility traits in the dataset used for analysis

| **Trait** | **No** | **Min** | **Max** | **Mean** | **SD** | **CV** |
| --- | --- | --- | --- | --- | --- | --- |
| AFC (days) | 183 | 567 | 1770 | 1168.14 | 182.56 | 15.00 |
| CI (days) | 370 | 310 | 855 | 480.38 | 115.77 | 25.00 |
| DO (days) | 364 | 45 | 560 | 194.32 | 114.51 | 57.00 |

AFC, age at first calving; CI, calving interval; DO, days open; N, number of records; Min, minimum; Max, maximum; SD, standard deviations; CV, coefficient of variation.

**Table S2** Additive, dominant, and allele substitution effects of SNPs in LAP3 and SIRT on fertility traits in Sahiwal and Karan Fries cattle

| **SNP** | **Genetic Effects** | **AFC (days)** | **CI (days)** | **DO (days)** |
| --- | --- | --- | --- | --- |
| rs717156555: C>G | Additive effect (a) | 564.5 | 232.0 | 91.5 |
|  | Dominant effect (d) | 630.5 | 243.0 | 96.5 |
|  | Substitution effect (α) | 924.8 | 377.8 | 149.4 |
| rs720373055: T>C | Additive effect (a) | 564.5 | 229.0 | 86.5 |
|  | Dominant effect (d) | 630.5 | 243.0 | 95.5 |
|  | Substitution effect (α) | 968.0 | 384.5 | 147.6 |
| rs516876447: A>G | Additive effect (a) | -57.5 | 0.50 | -1.0 |
|  | Dominant effect (d) | 3.50 | 1.50 | -5.0 |
|  | Substitution effect (α) | -57.1 | 0.68 | -1.6 |
| rs461857269: C>T | Additive effect (a) | 564.5 | 229.0 | 86.5 |
|  | Dominant effect (d) | 630.5 | 243.0 | 95.5 |
|  | Substitution effect (α) | 955.4 | 379.0 | 145.7 |
| rs720349928: G>A | Additive effect (a) | 564.5 | 229.0 | 86.5 |
|  | Dominant effect (d) | 630.5 | 243.0 | 95.5 |
|  | Substitution effect (α) | 968.0 | 384.5 | 147.6 |
| rs722359733: C>T | Additive effect (a) | -40.50 | 14.50 | 15.5 |
|  | Dominant effect (d) | 6.50 | 60.50 | 49.5 |
|  | Substitution effect (α) | -36.9 | 48.38 | 43.2 |
| rs462932574: T>G | Additive effect (a) | 564.5 | 231.5 | 86.5 |
|  | Dominant effect (d) | 630.5 | 243.5 | 95.5 |
|  | Substitution effect (α) | 968.0 | 387.3 | 147.6 |
| rs110932626: A>G | Additive effect (a) | -544.5 | -249.0 | -108.5 |
|  | Dominant effect (d) | 540.5 | 220.0 | 74.5 |
|  | Substitution effect (α) | -285.1 | -143.4 | -72.2 |
| rs43702363: C>T | Additive effect (a) | -546.0 | -251.0 | -111.0 |
|  | Dominant effect (d) | 536.0 | 219.0 | 72.0 |
|  | Substitution effect (α) | -256.6 | -132.7 | 72.0 |
| rs41255599:C>T | Additive effect (a) | 24.5 | 0.0 | 1.50 |
|  | Dominant effect (d) | -77.5 | 45.0 | 43.5 |
|  | Substitution effect (α) | 2.80 | 12.6 | 13.7 |
| rs718329990: T>C | Additive effect (a) | -52.0 | -30.5 | 20.0 |
|  | Dominant effect (d) | -59.0 | 45.5 | 47.0 |
|  | Substitution effect (α) | -61.4 | -23.2 | 27.5 |

**Table S3** Association of haplotype combinations of LAP3 gene promoter variants with fertility traits in dairy cattle

|  | **Least-square means ± standard error** | | |
| --- | --- | --- | --- |
| **Haplotype combination** | **AFC (days)** | **CI (days)** | **DO (days)** |
| H1H1(102) | 1066.36±27.28^b^ | 451.00±16.60^c^ | 167.00±15.70^b^ |
| H1H2(37) | 1180.80±45.11^ab^ | 467.00±23.40^c^ | 175.00±22.50^b^ |
| H1H3(44) | 1137.37±39.76^ab^ | 457.00±20.40^c^ | 170.00±19.30^b^ |
| H1H4(46) | 1114.50±36.69^ab^ | 449.00±19.40^c^ | 170.00±18.40^b^ |
| H2H2(43) | 1236.53±48.62^a^ | 445.00±22.60^c^ | 157.00±21.60^b^ |
| H2H3(35) | 1278.50±45.10^a^ | 561.00±24.0^a^ | 253.00±23.30^a^ |
| H2H4(35) | 1141.62±46.29^ab^ | 523.00±24.90^ab^ | 239.00±23.60^a^ |
| H3H4(28) | 1193.81±50.10^ab^ | 462.00±24.30^c^ | 173.00±23.0^b^ |
| *p* value | 0.0006^***^ | 0.00021^***^ | 0.0020^**^ |

Note: H means haplotype; the numbers in brackets represent the number of cows of the corresponding haplotype combination; H1H1 = CC-TT-AA-CC-GG-CC-TT, H1H2 = CC-TT-AG-CC-GG-CT-TT, H1H3 = CG-TC-AG-CT-GA-CC-TG, H1H4 = CC-TT-AG-CC-GG-CC-TT, H2H2 CC-TT-GG-CC-GG-TT-TT, H2H3= TC-GG-CT-GA-CT-TG, H2H4= TT-GG-CC-GG-CT-TT, H3H4 = CG-TC-GG-CT-GA-CC-TG. Least square means in the same column with different superscripts (a, b, and c) differ significantly at P < 0.001; Haplotypes with frequency less than 0.05 were ignored in analysis.
